# Methods for Evaluating the Effectiveness of Home Oral Hygiene Measures—A Narrative Review of Dental Biofilm Indices

**DOI:** 10.3390/dj11070172

**Published:** 2023-07-17

**Authors:** Gerarda D’Elia, William Floris, Lorenzo Marini, Denise Corridore, Mariana Andrea Rojas, Livia Ottolenghi, Andrea Pilloni

**Affiliations:** Section of Periodontics, Department of Oral and Maxillofacial Sciences, Sapienza University of Rome, #6 Via Caserta, 00161 Rome, Italy; gerarda.d.elia@gmail.com (G.D.); william7floris@gmail.com (W.F.); lorenzo.marini@uniroma1.it (L.M.); denise.corridore@uniroma1.it (D.C.); marianaandrea.rojas@uniroma1.it (M.A.R.); livia.ottolenghi@uniroma1.it (L.O.)

**Keywords:** biofilms, classification, dental caries, dental plaque, index, oral hygiene, periodontal diseases, periodontal index, preventive dentistry

## Abstract

Dental plaque is a biofilm structured in an extracellular matrix of polymers of host and microbial origin; the microorganisms can coexist in harmony with the host, thus guarantying oral health. Environmental modifications can lead to dysbiosis and onset of oral diseases; in fact, plaque is the etiological agent both of periodontal disease and dental decay. The use of an effective oral hygiene index should be considered as a relevant goal for the clinicians and the researchers, and consequently, numerous plaque indices have been proposed during the years. The present literature review aims primarily to obtain a complete summary of these scores to assess plaque deposits. It is useful because the clinician/researcher will select the right scoring method for the specific situation only if he knows the available options and if he is aware of both their strengths and weaknesses. This review applies a basic classification of plaque indices that distinguishes the ones that use non-quantitative methods from the ones that use quantitative methods. Non-quantitative methods are more subjective because they are based on the ability of the clinician to point out the presence or the entity of deposits, while quantitative methods introduce objectifiable means to measure plaque deposits.

## 1. Introduction

Dental plaque has been defined as a spatially organized and metabolically integrated community of microorganisms, i.e., as a biofilm structured in an extracellular matrix of polymers of host and microbial origin [1].

Plaque development is an articulated process, and it consists of phases of formation and maturation that occur over a period of weeks. The first event of the process is adhesion of bacteria to the salivary pellicle formed on teeth, thus allowing microorganisms to remain on surfaces despite the mechanical forces that tend to remove them. Due to the composition of this pellicle, streptococci are the pioneer species that can be found in the newly formed plaque; afterwards, later colonizers encounter and can bind other bacteria or molecules such as salivary mucins, agglutinin glycoproteins, glucans, and bacterial products. This mechanism is defined as “coadhesion”, and it allows plaque to welcome additional species with a greater proportion of Gram-negative bacteria, often potential periodontal pathogens [2].

Because of its behavior as a biofilm, dental plaque is composed by consortia of interacting microorganism that show greater capabilities than individual species. These amplified proprieties are represented by the possibility to create a heterogeneous environment suitable for the growth and the co-existence of different microbes, a very efficient metabolism, an enhanced resistance to stress and antimicrobial agents, and a stronger virulence (expressed, for example, in the pathogenesis of periodontitis) [3].

Under certain conditions, a relationship of harmony exists between the host and the microorganisms that form the microbiome, and a status of oral health can be observed. However, a great number of environmental modifications can occur, leading to dysbiosis and the onset of oral diseases [4,5]. In fact, it is well known that dental plaque is the etiological agent both of periodontal diseases and dental decay [6,7].

As for dental decay, pathogenesis is linked to the presence of microorganisms such as *Streptococcus mutans* that can produce acids following the exposition to dietary carbohydrates. A series of the phenomena of demineralization and remineralization occurs due to the oscillation of the pH of the oral cavity above and below the critical value of 5.0–5.5; when the balance between demineralization and remineralization is lost, caries formation is observed [6].

As for periodontal diseases, microorganisms present in the dental plaque play an etiological role in the pathogenesis of both gingivitis and periodontitis. An incipient dysbiosis causes a proportionate response by the immunological system of the host. This response is totally reversible, and it is represented by a series of non-specific inflammatory changes in the tissues that are defined as “plaque-induced gingivitis” [8]. When dysbiosis became frank, and the biofilm is pathogenic, the immune system of the susceptible host implements an exaggerated but ineffective response. This hyperinflammatory reaction leads to periodontitis and to its irreversible changes in the tissue, with loss of attachment, bone resorption, and pockets formation [9]. However, it must be remembered that the presence of specific microorganisms in the dental biofilm is indispensable for initiating the characteristic events of periodontitis, but it alone is not sufficient for the onset of the disease [10].

Plaque and calculus removal is therefore one of the principal goals of the therapy because it allows to prevent and to treat dental and periodontal diseases. However, to effectively reach this goal, a fundamental aspect is the tailoring of the maneuvers for biofilm control. During the years, great attention has been given by researchers to find new instruments capable of ideal characteristics, such as effectiveness, safety, reduced invasiveness, and maximum comfort [11]. The techniques for biofilm removal can be classified in professional and home approaches: the first ones are targeted at the elimination of both plaque and calculus from supragingival and subgingival environments, while the second ones are dedicated to removing plaque from the supragingival area. Examples of professional instruments are represented by powered devices (ultrasonic, sonic, and piezoelectric), hand instruments (as curettes and scalers), air-polishing, and rubber cup polishing. The most common home oral hygiene devices are represented by toothbrushes (both powered and manual) and interproximal instruments (such as dental floss and interdental brushes). Along with these mechanical instruments, there are also agents such as toothpaste and mouthwashes that can provide chemical plaque control [12].

The above-mentioned cause–effect connection between biofilm presence and dental and periodontal diseases explains the relevance of evaluating the oral hygiene status of patients through the introduction of plaque indices. Therefore, obtaining an effective oral hygiene index should be considered as a relevant goal for the clinician. For this purpose, many indices have been proposed and modified during the past years [13].

The present literature review aims primarily to obtain a complete summary of these non-quantitative and quantitative methods to assess oral/dental biofilm.

## 2. Assessment of the Oral and Dental Biofilm

### 2.1. Classification/Features of Plaque Indices

Indices can be classified based on different features, and this is of help for interpreting the data they report. A useful classification based on the objectivity of plaque assessment distinguishes between two separate categories: non-quantitative and quantitative methods. Non-quantitative indices rely completely on the skill of the clinician or the researcher to point out the presence and/or the entity of deposits; conversely, quantitative ones exploit objectifiable means to measure plaque deposits [14].

Additionally, indices can also be classified as “full mouth” or “simplified” depending on whether they measure the variable of interest in all sites or only in selected ones. Moreover, indices can measure a disease or other aspects of it as symptoms, treatment, or, as in the case of plaque indices, etiological factors. Finally, indices can be distinguished for their most suitable field of application: epidemiologic surveys, clinical trials, and evaluation of the progress of the patient and his/her motivation [15].

Plaque indices generally use the extent of the tooth area covered by plaque as the criterion for scoring. This extent can be evaluated in a subjective way by the clinician or, alternatively, by using photographs of teeth placed on grids with squares. Furthermore, some indices use the thickness of plaque deposits or their weight for the classification. For greatest success, an index should have minimal intra- and inter-examiner variation; to obtain this goal, the criteria should be well defined and the examiners well trained. Moreover, in order to maximize the reliability, in the recent years, great attention has been given to the introduction of more objective and reproducible ways for assessing plaque.

Plaque indices that require the scoring of all surfaces of all teeth should be preferred in every situation they can be applied. Alternatively, partial plaque indices are available; among them, the most adequate are based on the examination of six selected teeth (called Ramfjord teeth) that are quite representative of the full-mouth status [16].

### 2.2. Disclosing Agents

The first requirement to assess the oral hygiene status of a patient is detecting plaque, and a preliminary distinction among plaque indices can be performed according to the method used for this aim. In fact, some indices are based on observation with the naked eye of teeth surfaces, while other ones need the adoption of auxiliary means; passing an explorer or a probe over teeth is the simplest way to help the clinician to point out the presence of dental plaque, while applying disclosing agents is a more widespread method [17].

The mechanism of action of disclosing agents is due to the polarity differences between their molecules and the components of dental plaque, and consequently, they can interact and bind with each other, leading to a change of color in the biofilm, thus increasing the contrast with dental surfaces and allowing for easier identification of the deposits [18].

Thanks to the embedded property of such substances, dental plaque can be defined as a “stainable material” [19]. Iodine, Bismarck brown, erythrosine, methylene blue, basic fuchsin, and two-tone solutions are only some examples of the great variety of substances that have been studied and used since the early 20th century to make plaque evident [20].

The application of disclosing agents has two essential benefits: first of all, it facilitates the evaluation of plaque indices; secondly, it improves plaque removal both in clinical and home settings, helping to motivate the patient, to provide personalized instructions, and to raise awareness of the need for applying them [21,22].

### 2.3. Non-Quantitative Methods

Non-quantitative methods assess the presence of plaque without measuring it in an objective way. The result is a graduated scale delimited by a minimum and a maximum numerical value. The use of non-quantitative methods makes plaque indices easy to use both in clinical practice and in the research field. Moreover, they are advantageous because they do not require particular instruments to evaluate and measure plaque.

**Figure 1:**
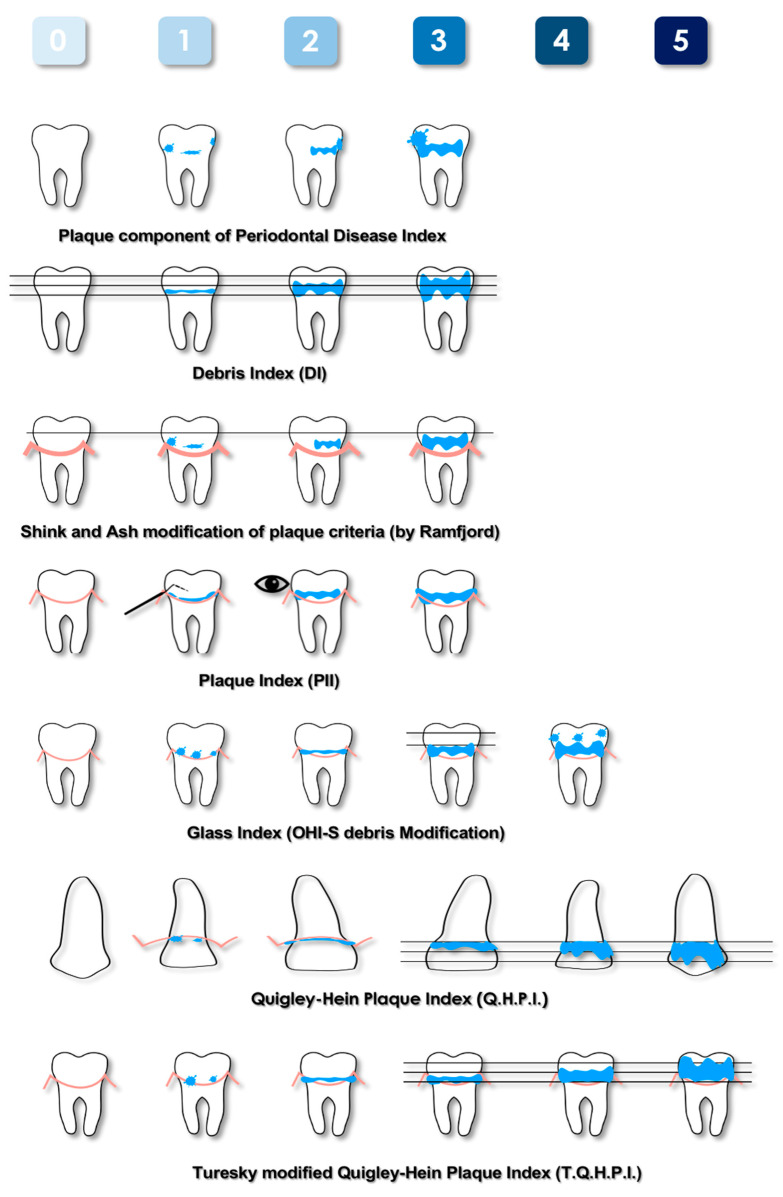

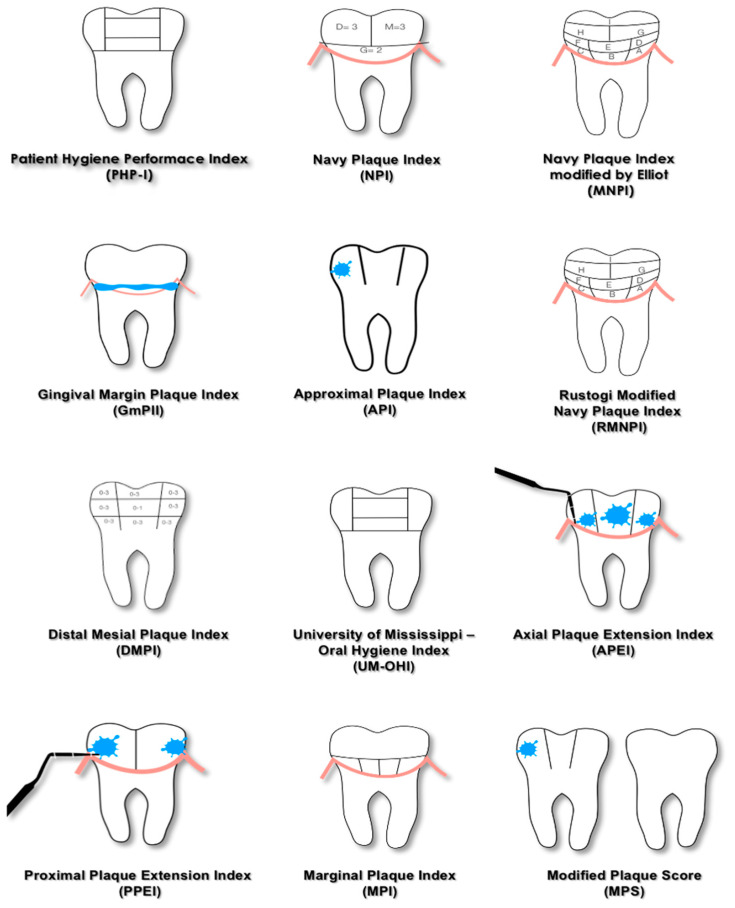
Schematic representation of the most-used plaque indices based on non-quantitative methods. Pink lines represent the gingival margin, blue areas represent plaque deposits.

Plaque indices based on non-quantitative methods described in the literature and their main features are listed in Table 1. Moreover, the most-used non-quantitative plaque indices are schematically represented in Figure 1.

#### 2.3.1. Area Measurements—Numerical Indices

The Plaque Component of the Periodontal Disease Index was proposed by Ramfjord in 1959 [16]. This index is not time-consuming, as it requires the evaluation of only six teeth. Despite being limited to a part of the dentition, it evaluates both the posterior and anterior teeth. Therefore, an overall assessment of the entire mouth can be generalized. Subsequently it was modified by Shick and Ash [24], who further reduced the evaluation to the buccal and lingual surfaces and to the gingival half of each tooth. The latter aspect was justified by the greater importance of this area in periodontal diseases. Due to the characteristics described, the Plaque Component of the Periodontal Disease Index and its modification can be considered for use in clinical studies aimed at evaluating the efficacy of agents or procedures that modify the development of plaque and its relationship with gingival pathologies.

In 1960, Green and Vermillion introduced the Oral Hygiene Index (OHI) [23]. This index is given by the sum of the Debris Index (DI) and Calculus Index (CI). The calculation needed to obtain the final score and the score of each sextant represented only by the tooth with the highest value were the major limits of this index. To overcome the complexity of this index, the Simplified Oral Hygiene Index (OHI-S) and the Simplified Debris Index (DI-S) [26] were proposed, which evaluate a smaller number of teeth and surfaces that are considered the most representative of the status of the patients. A further modification was proposed by Glass in 1965 [28], which allowed quantitation of differences within the gingival third area. The DI, the DI-S, and the Glass Index evaluate soft foreign matter loosely attached to the teeth, consisting of a mixture of bacterial plaque, food debris, and materia alba. For this reason, these indices could be indicated for epidemiological studies where subjects are not allowed to vigorously rinse to eliminate this loosely attached material before evaluation. Conversely, their application in clinical trials is more limited.

In 1962, the Quigley and Hein Plaque Index (QHPI) was presented [25]. This weighted score takes into account the subtle differences in plaque accumulation in the gingival third of the tooth. This feature makes this index highly valued, as it reflects the actual plaque–gingival inflammatory relationship. However, the evaluation of only the buccal surfaces of the anterior teeth is its major limitation. In fact, it can underestimate the real state of oral hygiene of the patient. For this reason, in 1970, it was modified by Turesky [30], who extended the evaluation to both the buccal and oral aspects of all teeth except the third molars. It has since become one of the most used indices, and it is recommended for clinical trials. Otherwise, its application in daily clinical practice is considered impractical.

The Navy Plaque Index (NPI), proposed in 1973 [33], scores the presence of plaque on six teeth, and each of them is divided into four areas. Despite its complexity and its limited clinical application, the present index was modified, and two new indices were introduced. The Navy Plaque Index modified by Elliott (MNPI) [32] and Rustogi Modified Navy Plaque Index (RMNPI) [43] require the further partition of dental surfaces; consequently, each of them is dived into nine areas. The areas of the tooth adjacent to the gingival margin are the smallest ones, thus emphasizing the presence of deposits in contact with the soft tissues because it plays an important role in the development of inflammation.

#### 2.3.2. Gingival Plaque Thickness

The main index that uses the thickness of the deposits as a criterion for evaluation is the Plaque Index (PlI) [27]. In its original formulation, PlI requires the observation of only six selected teeth; subsequently, its use as a full-mouth index was introduced.

The PlI is useful in the clinical field because it records the thickness of plaque deposits along the gingival margin, i.e., where they are more influent on the developing of inflammation. The main disadvantages of this index are the difficulty in detecting thin deposits with the naked eye and the time required because of the need of drying the surfaces to perform an accurate assessment. Moreover, the evaluation is quite subjective, as indicated by the terms such as “film”, “moderate”, and “abundance” used to describe the deposits; consequently, to reduce the variability, a single trained examiner should score the PlI when it is used for clinical trials, and it could be used in conjunction with other indices.

#### 2.3.3. Dichotomous Indices

After the introduction of the Plaque Control Record by O’Leary in 1972 [31], different dichotomous indices have been developed. The success of these indices is due to the fact that the principle of presence/absence is an easy and fast way to score plaque; moreover, this kind of evaluation is very useful for patient motivation both at baseline and during the follow-up visits. The Plaque Control Record and other similar dichotomous indices can also be used to calculate a Full-Mouth Plaque Score (FMPS), which is given by the percentage of sites with plaque on the total number of sites evaluated and expresses the oral hygiene status of the patient through a single value.

The above-mentioned aspect of education and motivation of the patient plays a central role for therapeutic success. It is well known that the goal of periodontal therapy is the control of disease as evaluable through clinical indices such as Probing Depth (PD), Bleeding on Probing (BoP), and Clinical Attachment Level (CAL). The improvement of the clinical status of the patient is more easily achieved and more predictably maintained when only a limited quantity of plaque is present [55]. A FMPS of 20–25% has been indicated as an acceptable threshold value because it is associated with the possibility of maintaining periodontal health and good surgical results both in the short and long term [56]. However, tighter plaque control is required to successfully perform regenerative periodontal surgery because lower levels of biofilm deposits are associated with greater amounts of clinical attachment gain. In this clinical situation, the maximum FMPS threshold value that is considered acceptable is 15%, and plaque should not be present at the surgical site [57].

A subclassification of non-quantitative plaque indices is proposed based on the method used for recording, as shown in Table 2.

### 2.4. Quantitative Methods

Despite the adjunctive strategies used to identify dental plaque, the non-quantitative methods share a fundamental limitation: they are based on the clinician’s capability to observe and assess the data, and therefore, they are subjective. In order to overcome this problem and to increase objectivity and reproducibility, quantitative indices were developed. Plaque indices based on quantitative methods described in the literature and their main features are listed in Table 3.

#### 2.4.1. Dental Plaque Weight

Weight was probably the first feature that was considered for developing a quantitative plaque index. The measurement of wet plaque weight was initially proposed [58] but did not prove to be a reliable method because of the evaporation of water, so this parameter was replaced by dry weight [59,60] without obtaining additional advantages over non-quantitative indices.

#### 2.4.2. Planimetric Indices

Planimetric indices are based on the analysis of dental images taken with different techniques and tools. The aim of these methods is the calculation of the extension of dental surfaces covered by plaque [61,62,63,64]. The first-introduced method used grids and diagrams and required human intervention to identify plaque, which was extremely time-consuming. The most recent methods utilize photographs that undergo digital analysis processes, requiring complex systems to obtain repeatable images with the use of specially designed devices. These methods are detailed in Table 3.

#### 2.4.3. Quantitative Light-Induced Fluorescence for Plaque Detection

Quantitative light-induced fluorescence (QLF) is a method used for the observation and assessment of dental surfaces that is based on the natural fluorescence of the teeth under certain conditions of light [65]. Some studies demonstrated a red/orange fluorescence of plaque deposits due to the porphyrins produced by microorganisms, and this feature is further enhanced using disclosing agents. Thanks to this finding, a new category of quantitative plaque indices was proposed, and a series of trials was conducted to prove their reliability [19,66,67,68].

#### 2.4.4. Automated Methods

Automated methods refer to a group of plaque indices that uses algorithms and software to identify the presence of plaque on digital photographs showing dental surfaces. Different features of plaque can be highlighted by the above-mentioned software, and some examples are listed below: its fluorescence when it is disclosed with fluorescein and illuminated by UV light [69]; the values of RGB (red, green, and blue) and HSI (hue, saturation, and intensity) of each pixel that makes up the photograph [70]; and the prevalence of “yellow” or “not yellow” in each pixel [71].

#### 2.4.5. Three-Dimensional Coordinates for Plaque Quantification

The last sub-group is represented by plaque quantification using 3D coordinates; this method was proposed by Yeganeh and co-workers, and it is based on the digitalization and comparison through a coordinate-measuring machine (CMM) of two impressions, with one taken before and the other after plaque removal is performed [72].

#### 2.4.6. Application of Quantitative Indices in Clinical Setting

Indices based on plaque weight and CMM also have a limited application in the research field due to their complexity and, for the first ones, their scarce accuracy. Consequently, considering adopt them in clinical setting is not so presumable. On the other hand, planimetric (those that do not require human intervention) and automated methods and biofilm detection using QLF could more realistically represent a future option for scoring plaque also during daily clinical activity.

The introduction of these new methods is obviously linked to the availability of modern instruments that allow to objectify the presence of biofilm deposits. The feasibility of adopting these new indices is consequently tied in a tight way to the possibility to dispose of the above-mentioned modern instruments.

Automated and planimetric indices exploit digital software capable of analyzing pictures taken in compliance with specific criteria in order to be reproducible and to emphasize the identified features by the software to highlight the presence of plaque. The limitation of the use of such indices is that making this kind of picture can be time-consuming, and often, dedicated equipment is needed; moreover, the clinician should be skilled at using the software.

In addition, QLF requires digital software for the analysis of pictures taken with specific devices, so it shares the same practical limitation of automated and planimetric indices. However, it must be considered that, beyond plaque scoring, QLF has the ability to detect demineralization of the tooth, allowing early caries diagnosis. Therefore, the clinician who wants to adopt modern methods in his/her practice could opt for QLF to improve with a single economic investment and learning curve both the ability of scoring plaque and diagnosing caries.

A subclassification of quantitative indices according to the parameters used for recording the biofilm deposits (i.e., extension, weight, thickness and fluorescence) is shown in Table 4.

## 3. Conclusions

The most common uses of plaque indices are the following: (i) the evaluation of the cleansing efficacy of a device or a product and (ii) the assessment of the relationship between deposits and periodontal health and/or dental caries.

The availability of a great number of plaque indices allows to choose the most suitable index for each purpose and situation. For example, indices that measure severity are extremely useful for epidemiologic surveys and clinical trials; on the other hand, dichotomous indices are particularly appropriate for clinical practice.

According to this wide choice, it is important to underline that when different plaque indices are used, only general findings can be compared. Finally, indices alterations should be avoided because they also require a change in the interpretation of data.

The researcher or clinician should be aware of both strengths and weaknesses of different methods for scoring plaque, and he/she should be able to optimize their features with the specific field of application. Selecting the right plaque index is a goal that can be obtained only through the knowledge of the various options; however, all indices should satisfy some ideal aspects independently in the specific field of application. These ideal aspects are listed below: (a) the index should be simple to use; (b) it should require minimal time and minimal instruments to perform the scoring; (c) the score criteria should be clearly explained to ensure maximum reproducibility and standardization; (d) the index should allow performing statistical analysis; and (e) the index should be equally sensitive throughout the scale.

Due to their great number and to the specific needs of both the clinical and research context, it is not easy to draw conclusions about the popularity of plaque indices. Generally, it can be stated that the most-used indices are the ones that assess the presence of plaque dichotomously and the ones that emphasize the presence of biofilm along the gingival margin. On the other hand, use of plaque indices that require complex processes for the assessment or specific devices is more limited.

## Figures and Tables

**Table 1:** Non-quantitative methods to assess the presence of dental plaque.

Name (Abbreviation)	Authors, Year	Soft Tissue Area or Teeth Examined (FDI Notation)	Aspects or Surfaces Examined	Disclosing Agents	Presence or Severity Measured	Formula
Plaque component of Periodontal Disease Index	Ramfjord, 1959 [16]	1.6, 2.1, 2.4, 3.6, 4.1, 4.4	All surfaces	Yes	0: No plaque; 1: plaque present on some but not all interproximal buccal and lingual surfaces; 2: plaque present on all interproximal, buccal, and lingual surfaces but covering less than one-half of these surfaces; 3: plaque extending over all interproximal buccal and lingual surfaces but covering more than one-half of these surfaces.	Scores for each surface are totaled and divided by the number of surfaces scored.
Debris Index (DI)	Greene and Vermillion, 1960 [23]	The tooth with the greatest amount of plaque deposits for each sextant (=segment)	Buccal, oral	No	0: No debris or stains; 1: soft debris covering not more than one-third of the tooth; 2: soft debris covering more than one-third but not more than two-thirds of the tooth; 3: soft debris covering more than two-thirds of the tooth.	Scores are totaled and divided by the number of segments scored.
Oral Hygiene Index (OHI)	Greene and Vermillion, 1960 [23]		-	No	-	Sum of DI and Calculus Index (CI).
Shick and Ash modification of plaque criteria (by Ramfjord)	Shick and Ash, 1961 [24]	All teeth	Buccal, oral (iuxtagingival half)	Yes	0: Absence of plaque; 1: presence of plaque covering less than one-third of the gingival half of the buccal surface; 2: presence of plaque covering one-third or less than two-thirds of the gingival half of the buccal surface; 3: presence of plaque covering two-thirds or more of the buccal surface. The same scoring system was used for the oral surface.	Sum of all buccal and oral scores is calculated and then divided by the maximum possible score. The result is then multiplied by 100 to obtain a percentage index.
Quigley–Hein Plaque Index (QHPI)	Quigley and Hein, 1962 [25]	1.3 to 2.3 3.3 to 4.3	Buccal, oral	Yes	0: No plaque; 1: flecks of stain at gingival margin; 2: definite line of plaque at gingival margin; 3: gingival third of surface; 4: two-thirds of surface; 5: greater than two-thirds of surface. This recording is performed for each surface.	Sum of the scores is divided by the number of examined surfaces, thus obtaining an average score.
Simplified Debris Index (DI-S)	Greene and Vermillion, 1964 [26]	Upper jaw: the first permanent teeth distal to the second bicuspid (usually the 1.6 and 2.6 ) Lower jaw: the first permanent teeth distal to the second bicuspid (usually the 3.6 and 4.6 ) 1.1 and 3.1.	Buccal Oral Buccal	No	0: No debris or stains; 1: soft debris covering not more than one-third of the tooth; 2: soft debris covering more than one-third but not more than two-thirds of the tooth; 3: soft debris covering more than two-thirds of the tooth.	The sum of the values is divided by the number of observed surfaces.
Simplified Oral Hygiene Index (OHI-S)	Greene and Vermillion, 1964 [26]	-	-	No	-	Sum of DI-S and Simplified Calculus Index (CI–S).
Plaque Index (PlI)	Silness and Löe, 1964 [27]	1.6, 2.1, 2.4, 3.6, 4.1, 4.4, or all teeth	Mesial, distal, facial, lingual	No	0: No plaque in the gingival area; 1: a film of plaque adhering to the free gingival margin and adjacent area of the tooth, where plaque may only be recognized by running a probe across the tooth surface; 2: moderate accumulation of soft deposit is within the gingival margin, which can be seen by the naked eye; 3: abundance of soft matter within the gingival pocket and/or on the gingival margin.	The sum of the values is divided by the number of observed surfaces.
Glass Index (OHI-S debris Modification)	Glass, 1965 [28]	Same as OHI-S	Same as OHI-S	No	0: No visible debris; 1: debris visible at gingival margin but discontinuous and less than 1mm in height; 2: debris continuous at gingival margin and greater than 1mm in height; 3: debris involving entire gingival third of tooth; 4: debris generally scattered over tooth surface.	Same as OHI-S.
Patient Hygiene Performance Index (PHP-I)	Podshadley and Haley, 1968 [29]	Same as OHI-S	Same as OHI-S	Yes	The tooth surface is mentally divided into 5 areas: mesial, medial occlusal, medial central, medial gingival, and distal. 0: No plaque; 1: plaque in only 1 area; 2: plaque in 2 areas; 3: plaque in 3 areas; 4: plaque in 4 areas; 5: plaque in 5 areas.	The sum of the values is divided by the number of observed teeth.
Turesky modified Quigley–Hein Plaque Index (TMQHPI)	Turesky, Gilmore, and Glickman, 1970 [30]	All teeth except 3rd molars	Buccal, oral	Yes	0: No plaque; 1: separate flecks of plaque at the cervical margin of the tooth; 2: a thin continuous band of plaque (up to 1mm) at the cervical margin; 3: a band of plaque wider than 1mm but covering less than 1/3 of crown; 4: plaque covering at least 1/3 but less than 2/3; 5: plaque covering 2/3 or more.	The sum of the values is divided by the number of observed teeth.
Plaque Control Record	O’Leary, Drake, and Naylor, 1972 [31]	All teeth	Mesial, distal, facial, lingual	Yes	Presence/absence.	Number of sites with plaque/number of sites evaluated × 100.
Navy Plaque Index modified by Elliot (MNPI)	Elliot, Bowers, Clemmer, and Rovelstad, 1972 [32]	1.6, 2.1, 2.4, 3.6, 4.1, 4.4	Buccal, oral	Yes	Teeth are divided into 3 parts: gingival, middle, and occlusal. The central part is divided into 2 sections (medial and distal). The gingival part is divided into 3 sections (mesial, middle, and distal), with each having a small area not exceeding 1 mm adjacent the gingival tissue. A total of 9 sections are then evaluated for presence (1) or absence (0) of plaque.	The sum of the values is divided by the number of observed sections.
Navy Plaque Index (NPI)	Grossman and Fedi, 1973 [33]	1.6, 2.1, 2.4, 3.6, 4.1, 4.4	Buccal, oral	Yes	The facial surfaces are divided into 3 areas: gingival area (G), mesial proximal area (M), and distal proximal area (D). Plaque in contact with the gingival margin is scored as follows: area M and D=3, area G=2. If plaque is not in contact with gingival tissue but it is on any tooth surface, 1 point is added to the facial/lingual score.	NPI score of the patient: the highest for any of the six examined teeth. Total NPI score: the sum of the NPI score of the six examined teeth.
Plaque Index	Lenox and Kopczyk, 1973 [34]	All teeth	Mesial, distal, buccal, oral	Yes	Absence = 0; presence = 1.	Total number of surfaces with plaque is divided by total number of observed surfaces, and the result is multiplied by 100.
Gingival Margin Plaque Index (GmPlI)	Harrap, 1974 [35]	All teeth except 2nd and 3rd molars	Buccal	Yes	Presence/absence of plaque in contact with gingival margin. It is not applicable in case of severe gingivitis.	Number of sites with plaque/number of sites evaluated × 100.
Visible Plaque Index (VPI)	Ainamo and Bay, 1975 [36]	Teeth of 1st and 4th quadrant	Mesial, oral, buccal	No	Presence/absence	Number of sites with plaque/number of sites evaluated.
Hygiene Analysis Index (HAI)	Love, Ramirez, and Fultz, 1975 [37]	All teeth except 3rd molars	Mesial, distal, facial, lingual	Yes	Presence/absence	Total score: the sum of surfaces with plaque. Percent score: total score/number of observed surfaces × 100.
Approximal Plaque Index (API)	Lange, Plagmann, Eenboom, and Promesberg, 1977 [38]	All teeth	Mesio-buccal, disto-buccal, mesio-oral, disto-oral	No	Presence of plaque (1)/absence (0) of plaque in the interproximal area.	Percentage of sites with plaque over the total number of sites Evaluated.
Soparkar’s modification of TMQHPI	Lobene, Soparkar, and Newman, 1982 [39]	All teeth Except 3rd molars	Mesio-buccal, mid-buccal, disto-buccal, mesio-oral, mid-oral, disto-oral	Yes	Same as TMQHPI.	Sum of the scores is divided by the no. of surfaces.
Plaque-Free Surfaces Index	Coontz, 1983 [40]	All teeth	Mesio-buccal, Mid-buccal, disto-buccal, mesio-oral, mid-oral, disto-oral		For each area: presence = 1/ absence = 0.	Sum of plaque free surfaces/number of total observed surfaces × 100.
Distal Mesial Plaque Index (DMPI)	Fischman, Cancro, Pretara- Spanedda, and Jacobs, 1987 [41]	All teeth	Buccal, oral		Each dental surface is divided into 9 areas, which will receive a score from 0 to 3. The central portion, which is identified with the letter “R”, is evaluated dichotomously ( 0 or 1).	Percentage of sites without plaque over the total number of sites evaluated.
Plaque-Free Score Index	Grant, Stern, and Listgarten, 1988 [42]	All teeth	All axial surfaces	No	Presence/absence	Percentage of sites without plaque over the total number of sites evaluated.
Rustogi Modified Navy Plaque Index (RMNPI)	Rustogi, Curtis, Volpe, Kemp, McCool, and Korn, 1992 [43]	All teeth	Buccal, oral	Yes	Each surface is divided into 9 sections: 3 of them (A–C) are for the most gingival portion, 3 of them (D–F) are placed coronally to the gingival ones, 2 (G and H) are for the distal and mesial part of the central part of the tooth, and 1 (I) is for the occlusal area. For each section, plaque is scored as 0 if absent or 1 if present.	Sum of all the areas positive for dental plaque divided by the number of surfaces.
Van der Weiden’s modification of Plaque Index	Van der Weiden, Timmerman, Nijboer, Lie, and Van Der Velden, 1993 [44]	All teeth	Mesio-buccal, mid-buccal, disto-buccal, mesio-oral, mid-oral, disto-oral	Yes	Same as Plaque Index by Silness and Löe.	The sum of the values is divided by the number of observed surfaces.
Claydon and Addy’s modification of Rustogi Modified Navy Plaque Index	Claydon and Addy, 1995 [45]	All teeth	Buccal, oral	Yes	Presence of plaque is recorded onto schematic diagrams of tooth surfaces; a transparent overlay with the same diagrams is then aligned with the clinical recording sheet. Each tooth diagram on the overlay has the A–I zones of the Navy Index modifications outlined. The scorer then decides whether the recorded areas coincided with each of the 9 zones.	Sum of all the areas positive for dental plaque divided by the number of surfaces. The index can be calculated as a full-mouth index, as a buccal index, or as an oral index.
University of Mississippi Oral Hygiene Index (UM-OHI)	Silberman, Le Jeune, Serio, Devidas, Davidson, and Vernon, 1998 [46]	All teeth	Buccal, oral	Yes	Each surface is divided into 5 areas, which are scored 0 if plaque is absent or 1 if it is present. The score of the entire surface is obtained by adding the scores of the areas. If plaque is present on both the interproximal areas, a capital P is added to the score. If plaque is present near the gingival margin, a capital G is added to the score.	The dentition is divided into 12 portions: 6 buccal sextants and 6 oral sextants. Each one of these portions receives the score of the tooth with the highest value within the sextant.
Occlusal Fissure Plaque Index	Addy, Renton-Harper, and Myatt, 1998 [47]	Premolars and molars	Occlusal	Yes	Presence of disclosed plaque in the fissure pattern of premolar and molar teeth and the extension of plaque out of the fissures to cover the occlusal surface.	A numerical index can be assessed using values from 0 to 5. An area index can be assessed considering the extension of disclosed plaque on a tooth grid.
Occlusal Site-specific Plaque Index	Levinkind, Owens, Morea, Addy, Lang, Adair, and Baron, 1999 [48]	Premolars and molars (unrestored)	Occlusal	Yes	The occlusal surfaces are divided by an imaginary grid into 9 zones for molars, 6 zones for upper 1st and 2nd premolars and lower 2nd premolars, and 4 zones for lower 1st premolars.	Score from 0 to 4 according to the perceived % plaque coverage of each zone.
Plaque Formation Rate Index (PFRI)	Axelsson, 2000 [49]	All teeth	All surfaces except occlusal ones	Yes	Presence of adherent plaque is recorded 24 hours after a professional cleaning; during this period, the patient must avoid any oral hygiene procedures.	Score from 0 to 5 according to the percentage of surfaces covered by plaque (0 = absence of plaque; 5 = high amount of plaque – i.e., over 40% of surfaces).
Proximal Plaque Extension Index (PPEI)	Matthijs, Moradi Sabzevar, and Adriaens, 2001 [50]	All teeth except incisors	Buccal, oral	No	Each surface is divided into 2 areas (mesial and distal). With the probe parallel to an imaginary diagonal placed perpendicular to the interdental papilla.	The extension of plaque for each point is recorded in millimeters (to the closest 0.5 mm).
Modification of the Quigley and Hein Index	McCracken, Heasman, Stacey, Steen, De Jager, and Heasman, 2002 [51]	All teeth	Mesio-buccal, mid-buccal, disto-buccal, mesio-oral, mid-oral, disto-oral	Yes	0: no sub- or supra-gingival plaque; 1: no supragingival deposits, subgingival plaque after sweeping ball tip along subgingival surface; 2: discrete deposits of supragingival plaque laterally along surface at the gingival margin; 3: continuous deposits of supragingival plaque extending less than 3 mm from the free gingival margin; 4: supragingival plaque extending coronally beyond 3 mm from the free gingival margin; 5: supragingival plaque extending coronally beyond 5 mm from the free gingival margin or extending to the occlusal surface/marginal ridge irrespective of the height from the gingival margin.	Full-mouth score calculated as the mean of all recorded values.
Dental Plaque Index (DPI)	Abe, Ishihara, Adachi, and Okuda, 2006 [52]	All teeth	Buccal, oral	No	DPI 0: no plaque; DPI 1: plaque covers less than half of the tooth surface; DPI 2: plaque covers more than half of the tooth surface.	DPI patient score is represented by the score of the area with the thickest deposit of plaque
Tongue Plaque Index (TPI)	Abe, Ishihara, Adachi, and Okuda, 2006 [52]	Tongue	-	Yes	TPI 0: absence of plaque; TPI 1: presence of plaque.	-
Marginal Plaque Index (MPI)	Deinzer, Jahns, and Harnacke, 2014 [53]	All teeth	Buccal, oral	Yes	The area close to the gingival margin is divided in four portions. For each portion, plaque is scored as 0 if absent or 1 if present.	Sum of all the portions positive for dental plaque divided by the number of portions.
Modified Plaque Score (MPS)	Park, Cho, and Han, 2018 [54]	1.6, 1.1, 2.7, 4.7, 3.1, 3.6	Buccal, oral	Yes	The buccal surface is divided in 3 parts (mesial, central, and distal), while lingual surface is evaluated as a whole. Each part or subpart can be scored from 0 to 3.	Sum of the scores is divided by the maximum score that can be obtained (72) × 100.

**Table 2:** Subclassification of non-quantitative plaque indices based on the method used for the recording.

Indices that record the extension of plaque using a numerical scale of values	Periodontal Disease Index (plaque component) [16] Debris Index (DI) and Oral Hygiene Index (OHI) [23] Shick and Ash modification of plaque criteria (by Ramfjord) [24] Quigley–Hein Plaque Index (QHPI) [25] Simplified Debris Index (DI-S) and Simplified Oral Hygiene Index (OHI-S) [26] Plaque Index (PlI) [27] Glass Index (OHI-S debris Modification) [28] Turesky modified Quigley–Hein Plaque Index (TMQHPI) [30] Navy Plaque Index (NPI) [33] Soparkar’s modification of TMQHPI [39] Distal Mesial Plaque Index (DMPI) [41] Van der Weiden’s modification of Plaque Index [44] Modification of the Quigley and Hein Index [51] Dental Plaque Index (DPI) [52] Modified Plaque Score (MPS) [54]
Dichotomous indices	Patient Hygiene Performance Index (PHP-I) [29]
	Plaque Control Record [31]
	Navy Plaque Index modified by Elliot (MNPI) [32]
	Plaque Index [34]
	Gingival Margin Plaque Index (GmPlI) [35]
	Visible Plaque Index (VPI) [36]
	Hygiene Analysis Index (HAI) [37]
	Approximal Plaque Index (API) [38]
	Rustogi Modified Navy Plaque Index (RMNPI) [43]
	Claydon and Addy’s modification of Rustogi Modified Navy Plaque Index [45]
	University of Mississippi—Oral Hygiene Index (UM-OHI) [46]
	Marginal Plaque Index (MPI) [53]
Indices that record plaque absence	Plaque-Free Surfaces Index [40]
	Plaque-Free Score Index [42]
Indices that record plaque in sites different from the axial surfaces of the tooth	Occlusal Fissure Plaque Index [47]
	Occlusal Site-specific Plaque Index [48]
	Tongue Plaque Index (TPI) [52]
Indices that evaluate the process of plaque formation	Plaque Formation Rate Index (PFRI) [49]
Indices that record plaque extension in millimeters	Proximal Plaque Extension Index (PPEI) [50]

**Table 3:** Quantitative methods to measure dental plaque.

Name (Abbreviation)	Authors, Year	Method	Teeth and Surfaces Examined	Measurement
Planimetric Plaque Index by Plüss et al.	Plüss, Engelberger, and Rateitschak, 1975 [61]	Planimetric index	Buccal surfaces of lower central and lateral incisors	After using a disclosing agent, a photo of the teeth is taken and superimposed with a grid. It is then calculated how much grid unit corresponds to the area of the teeth covered by plaque.
Wet Plaque Weight	Gilmore and Clark, 1975 [58]	Dental plaque weight	N/A	The method is based on the weight of the “wet” plaque.
Objective Quantification Method for Measuring In Vivo Accumulated Dental Plaque	Trapp, Noble, Navarro, and Green, 1975 [59]	Dental plaque weight	N/A	A gold insert is set on a crown of a posterior tooth. Once removed, the insert is placed in an oven at 95 °C for one hour and then cooled for 15 min with the aim of “drying” plaque. The insert weight is then compared to the initial weight to quantify plaque accumulation.
Photogrammetric registration of dental plaque accumulation in vivo	Bergström, 1980 [62]	Planimetric index	Buccal surfaces of the upper lateral incisors	After using a disclosing agent, two color photos (with slightly different positions) are taken using a special device equipped with a stereomicroscope and a plate that allows the patient to be positioned in order to make the photos repeatable. The images are subjected to a photogrammetric analysis and combined to form a three-dimensional model. The outline of the tooth and the area covered by the highlighted plaque are traced, and the system calculates the percentage of plaque.
Magiscan’s Plaque Measurement	Verran and Rocliffe, 1986 [63]	Planimetric index	Upper and lower anterior teeth (from canine to canine)	The teeth are photographed using a black and white film, a special flash, and a magnifying system recommended for intraoral images. The photographs are then enlarged. The outline of the teeth on an acetate sheet is drawn, and the limit of plaque highlighted with the disclosing agent is traced on a second acetate sheet. The sheets are placed one at a time under the “Magiscan” camera and transferred to a computer to measure the extent of traced areas. The ratio between the plaque area and the total area is then calculated and expressed as a percentage.
Plaque Percent Index (P% Index)	Söder, Jin, and Söder, 1993 [64]	Planimetric index	N/A	After using a disclosing agent, color images are taken with the camera perpendicular to the dental surfaces. Mirrors are used to take photos of the posterior teeth and of the oral surfaces of the anterior teeth. The images are studied using a system called CIAS (Computerized Image Analysis System), which calculates the number of pixels that make up the surface covered by plaque and the total tooth surface area, makes the ratio, and expresses it as a percentage.
Automatic Image Analysis (AIA)	Moradi Sabzevar, De Coster, and Adriaens, 1994 [73]	Automated method	N/A	A color photo is taken for each evaluated surface as perpendicular as possible to it. The photo is converted to black and white, and the contrast increased. Each surface is assigned a proportional score (% of the total surface covered by plaque).
Plaque thickness quantification using CMM	Yeganeh, Lynch, Jovanovski, and Zou, 1999 [72]	Plaque quantification using 3D coordinates	N/A	Two impressions are taken: one before and one after the removal of plaque; they are digitized by a laser probe connected to CMM. The two impressions are superimposed to evaluate the thickness of plaque at the gingival margin level.
Fluorescein disclosing and Digital Plaque Image Analysis (DPIA)	Sagel, Lapujade, Miller, and Sunberg, 2000 [69]	Automated method	N/A	After the application of the fluorescein, the photos are taken in suitable light conditions (UV). In the photo, dental plaque differs because it is yellow in color. The index is calculated as a percentage of pixels with plaque with respect to the total tooth surface.
Modification of the Plaque Percent Index	Staudt, Kinzel, Hassfeld, Stein, Staehle, and Dorfer, 2001 [74]	Automated method	Lingual surfaces	Reproducible photos are taken using a special camera positioner. A dedicated software is used to edit the photo and to calculate the percentage of pixels with plaque with respect to the total tooth surface.
Percentage Plaque Index (PPI) using QLF planimetric analysis	Pretty, Edgar, and Higham, 2004 [75]	Quantitative light induced fluorescence (QLF™)	Upper and lower anterior teeth (from canine to canine)	Digital photographs and images are acquired with a QLF device. They are evaluated through a planimetric analysis system. The number of pixels that constitute the total dental area and the number of pixels that constitute the area covered by plaque are calculated. Finally, the percentage index is obtained.
Hue Saturation Intensity (HSI) color space with the purpose of plaque detection	Carter, Landini, and Walmsley, 2004 [70]	Automated method	Upper and lower anterior teeth (from canine to canine)	Using methylene blue, a digital photo is taken. Thanks to a software, each of the three areas of interest (plaque, tooth, and gum) is divided into ten thousand pixels. For each pixel, the RGB and HIS values are calculated. The percentage plaque index is calculated.
Plaque detection with Quantitative Light Fluorescence (QLF)	Pretty, Edgar, Smith, and Higham, 2005 [19]	Quantitative light-induced fluorescence (QLF™)	Upper and lower anterior teeth (from canine to canine)	Two photos per tooth. Each photo is analyzed three times, and then, each tooth is rated six times to increase reliability. The photos are analyzed by a software that evaluates the areas with plaque based on three threshold values. The percentage index is then calculated.
Dry Plaque Weight	McCracken, Preshaw, Steen, Swan, deJager, And Heasman, 2006 [60]	Dental plaque weight	Interproximal surfaces between the distal aspect of the first premolar and the mesial aspect of the second molar, all quadrants	After drying with the air–water spray for 30 s, accumulated plaque is usually collected either from the area immediately below the contact point. Plaque samples are left at room temperature for one hour to completely evaporate the water and then weighed.
Occlusal plaque index proposed by Splieth and Nourallah	Splieth and Nourallah, 2006 [76]	Automated method	The occlusal surfaces of the molars	After using a disclosing agent, dental surfaces are photographed with an intraoral device. The images are analyzed using a conventional editing program to calculate the number of pixels that constitute the total surface and the number of pixels that constitute the surface covered by plaque. Finally, the ratio is calculated.
Analysis of dental plaque by using cellular neural-network-based image segmentation	Luan, Li, Kang, Liu, and Min, 2007 [77]	Automated method	Upper and lower anterior teeth (from canine to canine)	A photograph is taken and segmented with the cellular neural network (CNN) technique. This takes advantage of a gray threshold value to determine if the pixels correspond to the tooth surface with or without plaque.
Autofluorescence- based Plaque Quantification (APQ)	Han, Kim, Kwon, and Kim, 2015 [66]	Quantitative light-induced fluorescence (QLF™)	Buccal surfaces of upper and lower anterior teeth (from canine to canine)	A photo is taken with quantitative light-induced fluorescence—digital (QLF-D), a device that intensifies the intrinsic red fluorescence of plaque. The images are then analyzed to record a planimetric index that expresses the percentage of pixels with respect to the total number of pixels that constitute the tooth surface.
Image Analysis System (IAS) technique	Rosa and Elizondo, 2015 [78]	Automated method	Central incisors	Using erythrosine, photos are taken using a holder for the camera (to increase repeatability). In the first phase, the images can be viewed in 20× and cropped with an editing software in order to isolate the tooth from the other parts of the image. At this point, the red-colored areas (plaque) are automatically detected. Another software calculates the extension in mm^2^ of the tooth and the area covered by plaque, and then, the percentage value is calculated.
Simple Plaque Score (SPS) o QLF-D score	Lee, Choi, Mah, and Pang, 2018 [67]	Quantitative light-induced fluorescence (QLF™)	Full mouth, divided into 8 parts: upper and lower arch, right and left, front and back, buccal and oral surfaces	Images of these areas are collected using QLF-D and subjected to an analysis program. A score from 0 to 5 is assigned through the QA2 v1.23 program.
QLF-D ΔR score	Lee, Choi, Mah, and Pang, 2018 [67]	Quantitative light-induced fluorescence (QLF™)	Full mouth, divided into 8 parts: upper and lower arch, right and left, front and back, buccal and oral surfaces	Images of these areas are collected using QLF-D and subjected to an analysis program. According to the red fluorescence intensity, the following scores are assigned: ΔR30, ΔR70, and ΔR120. A higher ΔR value corresponds to a more intense red fluorescence and consequently to a greater degree of maturation of plaque.
Automated Digital Scoring System (ADSS) of Dental Plaque	Munro, Liang, Emechebe, Chen, Cairns, Manani, Hamilton, Good, and Kip, 2020 [71]	Automated method	Buccal and oral surfaces of all the teeth	Photos are taken, and they are adjusted so that they represent only the dental surfaces. Photos are analyzed by a special software that classifies each pixel as yellow (plaque) or not yellow (no plaque). The percentage value of the pixels with plaque is calculated with respect to the total pixels.
Fluorescent Plaque Index (FPI)	Park, Kahharova, Lee, Lee, de Josselin, de Jong, Khudanov, and Kim 2020 [68]	Quantitative light-induced fluorescence (QLF™)	Upper and lower anterior teeth (from canine to canine)	Photos are taken with a third generation QLF system. An algorithm evaluates the extent of the area covered by plaque and the intensity of the red fluorescence; a score from 0 (high level of oral hygiene) to 5 (low level of oral hygiene) is assigned.

**Table 4:** Subclassification of quantitative plaque indices based on the parameters used for the recording.

Indices that measure plaque extension using objective methods (planimetric indices and automated methods)	Planimetric Plaque Index by Plüss et al. [61]
	Photogrammetric registration of dental plaque accumulation in vivo [62]
	Magiscan’s Plaque Measurement [63]
	Plaque Percent Index (P% Index) [64]
	Fluorescein disclosing and Digital Plaque Image Analysis (DPIA) [69]
	Hue Saturation Intensity (HSI) color space with the purpose of plaque detection [70]
	Automated Digital Scoring System (ADSS) of Dental Plaque [71]
	Automatic Image Analysis (AIA) [73]
	Modification of the Plaque Percent Index [74]
	Occlusal plaque index proposed by Splieth and Nourallah [76]
	Analysis of dental plaque by using cellular neural network-based image segmentation [77]
	Image Analysis System (IAS) technique [78]
Indices that measure plaque weight	Wet Plaque Weight [58]
	Objective Quantification Method for Measuring In Vivo Accumulated Dental Plaque [59]
	Dry Plaque Weight [60]
Indices that measure plaque thickness using objective methods (3D coordinates for plaque quantification)	Plaque thickness quantification using CMM [72]
Indices that measure plaque using QLF™	Plaque detection with Quantitative Light Fluorescence (QLF) [19]
	Autofluorescence-based Plaque Quantification (APQ) [66]
	Simple Plaque Score (SPS) o QLF-D score [67]
	QLF-D ΔR score [67]
	Fluorescent Plaque Index (FPI) [68]
	Percentage Plaque Index (PPI) using QLF planimetric analysis [75]

## Data Availability

Not applicable.
